# Dual-Task Training Affect Cognitive and Physical Performances and Brain Oscillation Ratio of Patients With Alzheimer’s Disease: A Randomized Controlled Trial

**DOI:** 10.3389/fnagi.2020.605317

**Published:** 2020-12-22

**Authors:** Elnaz Parvin, Fatemeh Mohammadian, Sadegh Amani-Shalamzari, Mahdi Bayati, Behnaz Tazesh

**Affiliations:** ^1^Department of Exercise Physiology, Faculty of Physical Education and Sports Science, Kharazmi University, Tehran, Iran; ^2^Department of Neurology, Roozbeh Hospital, Tehran University of Medical Science, Tehran, Iran; ^3^Department of Exercise Physiology, Sports Medicine Research Center, Sport Sciences Research Institute, Tehran, Iran; ^4^Sports and Exercise Medicine Specialist, Roozbeh Hospital, Tehran University of Medical Sciences, Tehran, Iran

**Keywords:** aging, alpha wave, cognitive performance, dementia, electroencephalography, exercise, physical activity, theta wave

## Abstract

This study aimed to investigate the effect of 12 weeks of dual-task training on cognitive status, physical performance, and brain oscillation of patients with Alzheimer’s disease (AD). Twenty-six AD patients were randomly assigned to two groups, the training group (TG) and control group (CG). TG executed progressive combined exercises with visual stimulation twice a week for 12 weeks. Training included muscle endurance, balance, flexibility, and aerobic exercises with eyes closed and opened. Brain oscillation on electroencephalography (EEG) and a series of physical, cognitive, and mental tests were taken before and post-intervention. There was a significant improvement after training protocol in cognitive function, particularly in short-term and working memory, attention, and executive function (*p* < 0.01). Besides, there were substantial improvements in depression status (GDS scale), aerobic fitness (6 min walking), flexibility (chair sit and reach) functional ability (chair stand, timed up and go test), strength (knee extensions, preacher biceps curl, handgrip) in TG compared to CG. These signs of progress were associated with a significant increase (*p* < 0.05) in the frequency of brain oscillation and a decrease in the theta/alpha ratio. In addition to physical performance, the regular combined training with visual stimulation improves brain health as indicated by improving cognitive function and reducing the theta/alpha ratio.

**Clinical Trial Registration:** Iranian Registry of Clinical Trials (IRCT) https://www.irct.ir/, identifier IRCT20190504043468N1—August 5, 2020.

## Introduction

Alzheimer’s disease (AD) is a chronic neurodegenerative disease, without any known treatment. This disease progressively destroys brain structures, such as the hippocampus and entorhinal cortex, due to the accumulation of pathological forms of amyloid plaques and neurofibrillary tangles (Lane et al., 2018). Consequently, mental functions, including memory and cognition, are lost, leading to a decline in activities of daily living (McGough et al., 2017). In this regard, Burns et al. (2010) reported that reduced lean body mass in AD is associated with brain atrophy and declined brain function, including cognitive performance. In this regard, a positive correlation has been reported between Montreal Cognitive Assessment (MoCA) test and fitness parameters especially muscle strength (Tolea and Galvin, 2016; Xiao et al., 2020).

The electrical activity of the cerebral cortex (brain oscillation) can be recorded *via* electroencephalography (EEG) by placing electrodes on the scalp. The frequency of resting brain oscillation change in AD patients, compared to older individuals or those with mild cognitive impairments (MCIs), considering a decrease in alpha and beta power (Hsiao et al., 2013; Koelewijn et al., 2017) and an increase in theta power (Moretti et al., 2004; Hsiao et al., 2013). These changes are associated with altered cerebral blood flow, cognitive function (Lizio et al., 2011), and occipital gray matter density (Babiloni et al., 2015). Researchers demonstrate that alpha activity is strongly associated with working memory and probably with long-term memory (Başar, 2012; Başar and Güntekin, 2012). It seems that the brain oscillations ratio is important in relation to brain health. The theta/alpha ratio (Fahimi et al., 2017), which is a marker of AD and cognitive impairments, increases in patients with AD, compared to healthy individuals. A study reported a negative correlation (*r* = −0.52) between the theta/alpha ratio and the MoCA test in patients with type 2 diabetes (Bian et al., 2014). In patients with MCI, occipital alpha slowing may lead to AD (Babiloni et al., 2015). Also, the degree of reduction in alpha and beta peak frequencies is correlated with the stage of AD (Moretti et al., 2004; Koelewijn et al., 2017).

Epidemiological evidence suggests exercise training as a non-pharmacological approach to protect against AD (Rao et al., 2014; Huang et al., 2016; Jia et al., 2019; De la Rosa et al., 2020), increase the hippocampus size (Erickson et al., 2011), and increase brain neurogenesis (Liu and Nusslock, 2018). These structural changes are associated with functional improvements, such as improved independence and cognition of AD patients (Jia et al., 2019). Moreover, these exercise-induced brain changes are associated with alterations in the power of brain oscillation. However, to the best of our knowledge, no studies are investigating the effects of physical training on the frequency of brain oscillation in AD. In this regard, Jiang et al. (2019) reported that a 10-week limb exercise training leads to a significant increase in the alpha and beta wave power values in all brain areas of MCI patients which is associated with psychomotor speed and decline in cognitive function. Also, Gutmann et al. (2015) reported that the individual alpha peak frequency remained unchanged after 4 weeks of moderate exercise training in healthy individuals. Also, researchers have reported that acute bouts of exercise increase the power of beta oscillation in the frontal and central areas of the brain, which may indicate an increase in cortical activation (Moraes et al., 2007; Hubner et al., 2018); however, the long-term effects of physical training are unclear.

Researchers have shown that brain activation during exercise (a dual-task exercise) is beneficial for cognitive function (Brustio et al., 2018; Techayusukcharoen et al., 2019). Generally, training with eyes closed and remembering to do specific exercises with several stations are simple mental activities. In this regard, Hutt and Redding (2014) showed that an eyes-closed dance training increased the dynamic balance of ballet dancers, as closing the eyes led to a shift from visual to proprioceptive dependence for balance control. Moreover, researchers have found that closing the eyes activates different areas of the brain, especially the amygdala, which is involved in memory and learning (Marx et al., 2004; Lerner et al., 2009).

According to some researchers, unlike other oscillations, the power of alpha oscillations increases in a resting state with the eyes closed (Kan et al., 2017), whereas it differs when the person focuses on performing activities with the eyes closed. Dual-task exercises can be used to maintain the brain structure and function and improve physical independence in AD patients. Accordingly, eyes-closed exercises can activate the brain areas involved in memory to focus on activities; they may also increase alpha and beta oscillations (Barry et al., 2007).

Overall, AD causes impairments in different physical and mental functions. To the best of our knowledge, this is the first study to assess the effects of combined physical training with visual stimulation on the physical and cognitive functions of patients with AD. It is known that the power of brain oscillation reflects brain changes and that AD increases the theta/alpha ratio. Accordingly, we hypothesized that physical training combined with mental challenge could modify the power of brain oscillations. In this study, we aimed to investigate the effects of combined training with visual stimulation on the theta/alpha ratio, as well as the cognitive and physical health of patients with AD. Also, we aimed to explore the correlations between cognitive performance and fitness performance, as well as brain oscillations.

## Materials and Methods

### Study Design

This randomized clinical trial, with control and parallel groups, phase 2, and single-blind design was conducted on patients with AD. We aimed to investigate the effects of a 12-week dual-task training (low-intensity exercise with eyes open and closed), on the brain oscillation (alpha, beta, and theta), cognitive and physical performances of patients with AD. One week before the study, the participants and their caregivers attended three familiarization sessions, where they were informed about the benefits and potential risks of the study, signed a consent form, and participated in pretests. The block randomization method was applied before the study, and the participants were assigned to two groups, including the training group (TG) and the control group (CG). Brain oscillation, psychological and cognitive status, and physical fitness parameters, including body composition, aerobic capacity, muscle strength, flexibility, and functional abilities, were assessed in familiarization sessions.

### Participants

Patients with AD, who were eligible to participate in this study, were recruited from the memory clinic of Roozbeh Hospital in Tehran, Iran. AD patients, with mild dementia and the ability to walk and move independently, were included in this study. A neurologist confirmed the diagnosis of dementia, based on the Diagnostic and Statistical Manual of Mental Disorders (DSM-5) criteria. Brain imaging and laboratory tests were performed to exclude other causes of dementia. The AD severity was determined, based on the Functional Assessment Staging Test (FAST).

The patients’ medications, including choline esterase inhibitors (rivastigmine and donepezil), memantine, and selective serotonin reuptake inhibitors (SSRIs including sertraline, citalopram, and trazodone), were reviewed before recruiting the patients in the study. The medications were not changed during the intervention in terms of type or dosage. Before entering the study, all patients received cardiac consultation to rule out possible cardiac diseases or ischemia. Patients with serious cardiac diseases (e.g., unstable angina and recent myocardial infarct) were excluded.

**Figure 1:**
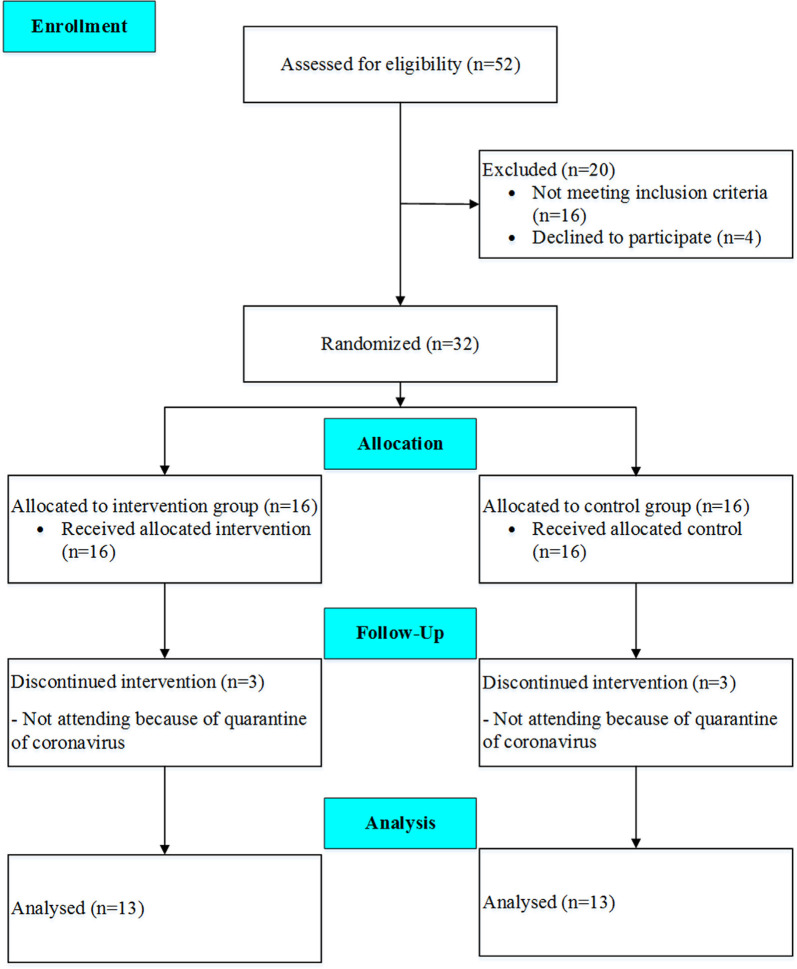
CONSORT flow diagram of the study.

Thirty-two eligible subjects volunteered to participate in the study, but the data of 26 patients (age: 67.4 ± 8.8 years; height: 165.8 ± 7.8 cm, body mass: 72.7 ± 11.3 kg, BMI 26.5 ± 4.3 kg/m^2^), who completed the pre- and post-tests, were finally analyzed. The participants were randomly assigned into two groups, including the TG and the CG. A CONSORT flow diagram of the present study is shown in Figure 1. On the other hand, the exclusion criteria were as follows: (1) deterioration of health condition; (2) inability to perform training; (3) lack of interest in continuing training; (4) not completing the posttest; and (5) the physician’s decision to exclude the participant from the study. To estimate the number of participants in each group, a sample size calculation was performed using G*Power Software version 3.1.9.6 (Faul et al., 2007) for repeated measure ANOVA, using a rejection criterion of 0.05 and 0.8 (1-beta) power, and large effect (*f* = 0.5), a minimum of 13 participants need to each group. All research procedures were approved by the Ethic committees for Sport Sciences Research Institute of Iran (approval number: IR.SSRI.REC.1398.037) and were conducted following the Declaration of Helsinki and reported according to CONSORT guidelines (Schulz et al., 2010). The study has been registered in the Iranian Registry of Clinical Trials (IRCT; one of the Primary Registries in WHO Registry Network) with registration number: IRCT20190504043468N1.

### Measures

Before and after training, the participants underwent a series of tests. All training sessions and tests were performed at Roozbeh Hospital Medical Center under the supervision of a sports medicine physician.

### Cognitive Status

The Montreal Cognitive Assessment (MoCA) test, developed by Nasreddine et al. (2005) for MCI and dementia, evaluates different domains of cognitive functioning. The reliability of this test was 92%, based on Cronbach’s alpha, and its internal consistency (IC) was 83% (Sikaroodi et al., 2012). The maximum score of the test is 30, with a score of 26 or higher considered to be normal. This test, which is executed within 10 min, includes different domains: short-term memory (five points); executive function, including Trail Making Test B, Clock Drawing Test, and visuospatial function test (cube copying; five points); attention and working memory (six points); language, including naming, repetition, and fluency (six points); abstraction (similarity; two points); and orientation to time and place (six points). Patients with scores of 26 or higher did not have any cognitive impairments (normal MoCA), whereas patients with scores lower than 26 probably had cognitive impairments.

### Depression Questionnaire

The Geriatric Depression Scale (GDS) was used to assess depression in the participants. In this questionnaire, all questions are of similar weight and have a yes/no response format. The maximum score of GDS is 15, and the minimum score is zero, with higher scores indicating more severe depression. This scale is one of the best tools for measuring depression in the elderly and patients with dementia. The sensitivity of 92% and specificity of 89% have been reported for this questionnaire (Bakhtiyari et al., 2014). The validity and reliability of 15-item GDS were measured by Malakouti et al. (2006) in Iran, and the best cut-off point was eight, with 90% sensitivity and 84% specificity (Sikaroodi et al., 2012).

The 15-item GDS captures depressive symptoms over the past week, using a yes/no response format. For 10 items, a positive response (“yes”) is given a score of one, and for five items, a negative response (“no”) is given a score of zero. Also, five items are reverse-scored (one for “no” and zero for “yes”). The total score of the items ranges from 0 to 15, with higher scores indicating more depressive symptoms. The GDS-15 score has been used as both continuous and categorical variables elsewhere. We used a cut-off score of ≥5 to indicate the presence of clinical depression symptoms (0, GDS-15 score <5 and 1, GDS-15 score ≥5). We also considered the continuous score of GDS-15 as the outcome (Cron et al., 2016; Honjo et al., 2018; Koohsari et al., 2019).

### Anthropometric Indices

Body composition indices, including height (stadiometer, Seca 213, Germany), body mass (digital weighing scales, Seca 769, Germany), body mass index (BMI; kg/m^2^), and body fat percentage (BF %; InBody S10, Biospace Company Limited, Seoul, South Korea), were assessed in this study.

### Maximum Strength

The maximum strengths of knee extensions, preacher curls, and handgrips were measured for all participants. One-repetition maximum (1-RM) for leg extensions and preacher curls was also determined, based on the procedures described by Sheppard and Triplett (Sheppard and Triplett, 2016). The participants performed a general warm-up, consisting of 5-min pedaling on a stationary bicycle (50–70 rotations per minute at a resistance level of 1–5), followed by a specific warm-up of two sets (5–20 repetitions at 40–50% of perceived maximal effort). Next, they made 3–5 attempts to reach 1RM, with 3–5 min of rest between attempts.

For knee extensions, the participants were asked to sit on a machine (Impulse IT95 Leg Extension, Impulse Health Tech Company Limited, Shandong, China). The researcher adjusted the chair in a way that the subject’s legs were placed under the pad, and his/her feet pointed to the pad while extending the knees. In preacher biceps curls, the participant adjusted the preacher bench, held a dumbbell with fully extended arms, and curled it up to shoulder level. Also, a grip strength dynamometer was used to measure the maximum isometric strength of the hand and forearm muscles. After adjusting the handle of the dynamometer for the subjects, they were asked to hold it in their hands, while keeping the arms at the right angles and the elbows on two sides of the body. Participants pressed the dynamometer with maximum isometric effort, which was maintained for about 5 s (Roberts et al., 2011). The best result of the three trials was recorded for each participant.

### Functional Tests

The timed up and go (TUG) and chair stand tests were used to measure functional abilities. The TUG test requires the participant to stand up from a chair without the use of arms, walk 2.4 m, turn, return to the chair, and sit (Bigdeli et al., 2020). Also, the chair stand test requires the participant seated on a chair to stand up as many times as possible within 30 s. The participants were instructed to keep their arms crossed at the wrists and hold them in front of the chest. The examiner counted the number of stands performed correctly within 30 s (Rikli and Jones, 2013). Chair “sit-and-reach”: (CSR) test requires the participant to sit on the edge of a chair, with one foot flat on the floor and the other leg extended forward with the knee straight and heel on the floor. By placing one hand on top of the other, the subject stretched his/her hands toward the toes by bending at the hip. Next, the distance between the tip of the fingertips and the toes was recorded as a score. If the fingertips reached the toes, the score would be zero; if the fingertips did not touch the toes, the score would be negative; and if the fingertips overlapped, the score would be positive. Overall, two trials were conducted for each participant, and the best distance was recorded (Bigdeli et al., 2020). The six-minute walk test (6MWT) was designed to assess aerobic fitness. In this test, the participants walked at a self-selected pace and were allowed to stop or change their pace (Rikli and Jones, 2013). In the indoor setting, two cones were placed 30 m apart, and the participants were asked to walk back and forth. The walking path was marked every 1 m to determine the distance accurately. For safety, a supervisor accompanied the participants.

### Brain Oscillation

Electroencephalography (EEG; SOMNO medics, SSP full EEG, Germany) was used to evaluate the brain oscillation with high sensitivity. The information related to beta, theta, and alpha changes on the EEG test, investigated by a neurologist, was used to determine the patient’s status. EEG was obtained over 10 min, and then, the percentage of each brain oscillation and the brain oscillation index were extracted, based on the visual scale. We also divided theta power by alpha power to calculate the theta/alpha ratio.

The 10-20 System which was recommended by the International Federation of Clinical Neurophysiology (IFCN; Deuschl and Eisen, 1999), were used in our study. Also, 21 channels of simultaneous recording are used to obtain EEG recording. In every case, an isolated ground electrode was placed between Cz and Pz.

Interelectrode impedances be checked as a routine prerecording procedure. In our study, impedances up to 10 kOhms are acceptable. Ten seconds of a square wave calibration were made before initiation of the recording in every patient. After that, a visual review of a 30-s run on the system reference montage without the notch filter. The sensitivity of our EEG system was set in 7 μV/mm of trace deflection. The low-frequency filter set in 1 Hz and the high-frequency filter set in 70 Hz to prevent artifacts or changes in electrode impedances that will negatively impact the quality of the EEG.

We record EEG recording at rest in 20 min and then choose 10 min of our recording which has a lower percentage of the artifacts (our patients due to background disease, dementia, had limited and poor cooperation compare to other patients and we should address this point in recording and analyze EEG recording). We reviewed the EEG in at least three different montages including two bipolar and one referential montage. Our recordings included periods when the eyes are open and when they are closed to review the effect of eye-opening on the attenuation of the alpha rhythm. A single-channel electrocardiogram (ECG) is included on one EEG channel.

All EEG recordings were performed in an awake state. According to significant cardiovascular risk factors in numerous dementia patients and patient inability to cooperate, Hyperventilation and Photic stimulation were not performed in patients with AD.

### Visual EEG Assessment

The certified clinical neurophysiologists, assessed the entire 20-min EEG recording by visual rating scale and according to a standardized visual rating scheme, which includes the severity of EEG abnormalities and the presence of focal, diffuse, and epileptiform abnormalities.

Source derivation was used as a reference (Hjorth, 1975), and the data was band-pass filtered in four frequency bands: delta (0.5–4 Hz), theta (4–8 Hz), alpha (8–13 Hz), beta (13–30 Hz). Oscillations >30 Hz were excluded from further analyses because of the expected artifacts from muscle and eye movement (Hagemann and Naumann, 2001).

### Training Protocol

The participants in the experimental group performed 24 workouts twice a week for 12 weeks. Each session lasted about 40–60 min, including 10 min of warm-up, 20–40 min of main exercises, and 10 min of cool down. The participants adhered to a combined protocol, including simple brain activities (eyes-closed training and cognitive activities) and physical activities (muscle endurance, balance, and aerobic capacity). The main training protocol consisted of five parts.

The first part of the training protocol included sitting and standing on an armchair, accompanied by shoulder girdle strengthening (three sets with 5–15 reps, followed by a gradual increase in resistance and repetition, using dumbbells and TheraBand). The second part included crossing over five sponge obstacles (height: 15–20 cm) with eyes closed (two repetitions in the first three sessions, gradually increasing to two reps every three sessions); the distance between the obstacles was variable. In the third part, the participants crossed over a safe balance beam board (2 m) with eyes closed (two repetitions in the first three sessions, gradually increasing to two reps every three sessions).

In the fourth part, six-vowel stations were placed in a semicircular arrangement at a 4-m distance in front of the subject with eyes closed. The subjects were asked to identify the sound of each station, move toward it, perform the predetermined exercises for 15 s (e.g., butterfly curls, Hercules curls, knee raises, hand raises, and biceps curls), and return. There were only two stations in the first session, which increased by one station every three sessions to reach a total of six stations. In the last part, there were four colored lights in front of the participants, each indicating a predetermined exercise. As long as the light was on (10–15 s), the subject was required to perform the relevant exercise (e.g., red light: side-right lunge; blue light: side-left lunge; green light: backward right lunge; and yellow light: backward left lunge). This part lasted for 2 min in the first session, which increased by 1 min every three sessions to reach 5 min by the end.

The exercises changed every three sessions and became more intense. The workouts were performed individually, and each individual attended the center at a certain time. The researcher accompanied the participants throughout the training. The intensity of training was difficult due to the variety of exercises. To monitor the workout intensity, heart rate (HR) was monitored by a smartwatch.

### Statistical Methods

Data presented in mean ± standard deviation (SD). The Statistical Package of Social Sciences (SPSS, IBM, v19) was used to analyze data. A repeated measure analysis of variance ANOVA with the time (T1 vs. T2) and protocol (TG vs. CG) was performed to analyze data. To assess the magnitude and direction of the linear correlations between the percentage change of the performance parameters and perceptual indices (MoCA and GDS), bivariate Pearson’s correlation coefficient (r) was calculated. Effect size (ES) was also computed as the change score divided by the SD of the change score to examine the magnitude of differences while controlling for the influence of the sample size (Dankel and Loenneke, 2018) with 0.2 considered as a small ES, 0.5 as a moderate ES and >0.8 as a large ES (Batterham and Hopkins, 2006). The significance level was set at *p* ≤ 0.05 for all statistical analyses. To determine the test-retest absolute and relative reliability, the coefficient of variation (CV) and intra-class correlation coefficient (ICC) was calculated. The ICC was calculated by a two-way single measure absolute agreement model and the CV was calculated by the formula (CV = [SD/mean] × 100). The CV for tests was <4.0% and ICC was >0.98. Figures were prepared in GraphPad Prism (Version 7.03, GraphPad Software).

## Results

### Cognitive Performance

**Figure 2:**
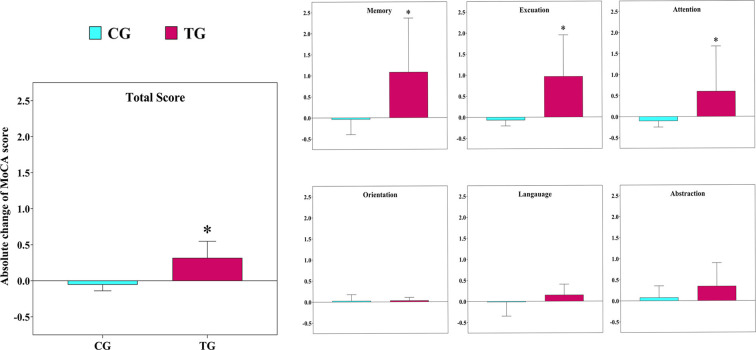
The absolute changes in the scores of the Montreal Cognitive Assessment (MoCA) test following the 12-week intervention in both groups. TG, training group; CG, control group. *The significant difference between groups.

The statistical analysis indicated there was a significant main group (between group; *F*_(1,12)_ = 13.5 *p* = 0.003, $\eta_{\text{p}}^{2}$: 0.53), time (within group; *F*_(1,12)_ = 28.1 *p* = 0.001, $\eta_{\text{p}}^{2}$: 0.70), and interaction effect (group × time; *F*_(1,12)_ = 40.5 *p* = 0.001, $\eta_{\text{p}}^{2}$: 0.77) for MoCA. In details, we observed a significant main group (*F*_(1,12)_ = 7.9 *p* = 0.016, $\eta_{\text{p}}^{2}$: 0.40), time (*F*_(1,12)_ = 5.0 *p* = 0.044, $\eta_{\text{p}}^{2}$: 0.30), and interaction effect (*F*_(1,12)_ = 13.6 *p* = 0.003, $\eta_{\text{p}}^{2}$: 0.53) for attention and working memory. We found no significant main group effect for the short-term memory (*F*_(1,12)_ = 3.2 *p* = 0.101, $\eta_{\text{p}}^{2}$: 0.21), through a significant time (*F*_(1,12)_ = 12.9 *p* = 0.004, $\eta_{\text{p}}^{2}$: 0.52) and interaction effect observed (*F*_(1,12)_ = 27.0 *p* = 0.001, $\eta_{\text{p}}^{2}$: 0.69). Also, we observed no significant main group (*F*_(1,12)_ = 0.1 *p* = 0.991, $\eta_{\text{p}}^{2}$: 0.01) for the executive function and visuospatial power, through a significant time (*F*_(1,12)_ = 22.8 *p* = 0.001, $\eta_{\text{p}}^{2}$: 0.66) and interaction effect existed (*F*_(1,12)_ = 38.8 *p* = 0.001, $\eta_{\text{p}}^{2}$: 0.76). However, for orientation, there were no significant main group (*F*_(1,12)_ = 0.6 *p* = 0.468, $\eta_{\text{p}}^{2}$: 0.05), time (*F*_(1,12)_ = 0.7 *p* = 0.436, $\eta_{\text{p}}^{2}$: 0.05), and interaction effect (*F*_(1,12)_ = 2.2 *p* = 0.165, $\eta_{\text{p}}^{2}$: 0.15). In addition, we found no significant main group (*F*_(1,12)_ = 4.5 *p* = 0.055, $\eta_{\text{p}}^{2}$: 0.27), time (*F*_(1,12)_ = 0.23 *p* = 0.636, $\eta_{\text{p}}^{2}$: 0.02), and interaction effect (*F*_(1,12)_ = 3.8 *p* = 0.075, $\eta_{\text{p}}^{2}$: 0.24) for language. Furthermore, there was no significant main group (*F*_(1,12)_ = 0.02 *p* = 0.901, $\eta_{\text{p}}^{2}$: 0.01), time (*F*_(1,12)_ = 3.3 *p* = 0.096, $\eta_{\text{p}}^{2}$: 0.21), and interaction effect (*F*_(1,12)_ = 1.9 *p* = 0.190, $\eta_{\text{p}}^{2}$: 0.14) for the abstraction (Figure 2).

### Psychological Status

There was no significant main group (*F*_(1,12)_ = 0.2 *p* = 0.631, $\eta_{\text{p}}^{2}$: 0.02), but a significant main time (*F*_(1,12)_ = 23.7 *p* = 0.001, $\eta_{\text{p}}^{2}$: 0.66) and interaction effect existed (*F*_(1,12)_ = 21.2, *p* = 0.001, $\eta_{\text{p}}^{2}$: 0.64) for GDS.

### Physical Performance

Descriptive statistics of performance and perceptual parameters pre- and post-intervention are summarized in Table 1. In overall, TG compare to CG demonstrated substantial improvements in all performance indices following a 12-week intervention. We found a significant main group (*F*_(1,12)_ = 6.4 *p* = 0.026, $\eta_{\text{p}}^{2}$: 0.35), time (*F*_(1,12)_ = 40.0 *p* = 0.001, $\eta_{\text{p}}^{2}$: 0.77), and interaction effect (*F*_(1,12)_ = 53.7 *p* = 0.001, $\eta_{\text{p}}^{2}$: 0.82) for 6 min walking. For chair sit and reach, there was no significant main group (*F*_(1,12)_ = 0.9 *p* = 0.342, $\eta_{\text{p}}^{2}$: 0.07), though a significant time (*F*_(1,12)_ = 87.6 *p* = 0.001, $\eta_{\text{p}}^{2}$: 0.88) and interaction effect existed (*F*_(1,12)_ = 135.9 *p* = 0.001, $\eta_{\text{p}}^{2}$: 0.92). Furthermore, following the 12-week intervention, we found a significant main group (*F*_(1,12)_ = 11.2 *p* = 0.006, $\eta_{\text{p}}^{2}$: 0.48), time (*F*_(1,12)_ = 80.2 *p* = 0.001, $\eta_{\text{p}}^{2}$: 0.87), and interaction effect (*F*_(1,12)_ = 61.3 *p* = 0.001, $\eta_{\text{p}}^{2}$: 0.84) for strength of preacher biceps curl. For strength of knee extensions, there also was a significant main group (*F*_(1,12)_ = 6.1 *p* = 0.030, $\eta_{\text{p}}^{2}$: 0.34), time (*F*_(1,12)_ = 25.1 *p* = 0.001, $\eta_{\text{p}}^{2}$: 0.68), and interaction effect (*F*_(1,12) =_ 38.2 *p* = 0.001, $\eta_{\text{p}}^{2}$: 0.76). For strength of handgrip, there was no significant main group (*F*_(1,12)_ = 2.3 *p* = 0.152, $\eta_{\text{p}}^{2}$: 0.16), but significant time (*F*_(1,12)_ = 63.6 *p* = 0.001, $\eta_{\text{p}}^{2}$: 0.84) and interaction effect observed (*F*_(1,12)_ = 74.2 *p* = 0.001, $\eta_{\text{p}}^{2}$: 0.86).

**Table 1:** Performance and psychological characteristics of participants pre- and post-intervention.

Variable	Group	Pre	Post	% change	Cohen’s *d*	*p*
6 min walking (m)	TG	177.0 ± 81.5	318.9 ± 85.5	96.9	1.9	0.001
	CG	180.0 ± 66.2	174.2 ± 62.9	−2.6	−0.5
Knee extension (kg)	TG	10.8 ± 5.6	23.1 ± 10.6	134.6	1.6	0.001
	CG	10.7 ± 4.9	10.0 ± 4.5	−4.5	−0.6
Biceps curl (kg)	TG	6.4 ± 1.7	10.6 ± 2.5	70.2	2.6	0.001
	CG	6.4 ± 1.6	6.1 ± 1.5	−3.1	−0.3
Handgrip (kg)	TG	23.1 ± 9.1	31.6 ± 8.9	47.9	2.4	0.001
	CG	22.61 ± 8.3	21.3 ± 8.1	−7.1	−1.4
30 s stand-up (N)	TG	10.3 ± 3.4	18.5 ± 0.37	94.8	1.9	0.001
	CG	9.7 ± 3.1	9.2 ± 2.3	−2.7	−0.5
Timed Up and Go test (s)	TG	11.8 ± 2.5	6.4 ± 1.4	−45.6	−3.5	0.001
	CG	11.8 ± 2.7	12.4 ± 2.9	5.7	0.8
Chair sit and reach (cm)	TG	18.5 ± 8.1	26.9 ± 7.6	54.5	3.9	0.001
	CG	19.8 ± 8.1	18.7 ± 7.1	−3.7	−0.5
MoCA	TG	18.6 ± 3.5	23.9 ± 2.3	28.4	1.7	0.001
	CG	19.0 ± 2.1	17.9 ± 2.2	−3.3	−0.5
GDS	TG	5.4 ± 2.9	2.6 ± 1.9	−49.5	−1.4	0.001
	CG	4.4 ± 2.5	4.5 ± 2.5	6.8	0.2
Alpha oscillation (%)	TG	80.0 ± 5.5	83.2 ± 2.7	4.3	0.7	0.002
	CG	78.8 ± 7.7	78.0 ± 7.6	−1.0	−0.6
Beta oscillation (%)	TG	3.7 ± 3.1	8.9 ± 3.3	218.3	2.6	0.001
	CG	3.1 ± 1.6	3.0 ± 1.1	5.0	−0.2
Theta oscillation (%)	TG	16.2 ± 6.5	7.8 ± 3.7	−51.8	−1.9	0.001
	CG	18.1 ± 6.9	19.0 ± 7.0	5.9	0.6

*TG, training group; CG, control group; MoCA, the montreal cognitive assessment; GDS, geriatric depression scale*.

For functional indices, we found a significant main group (*F*_(1,12)_ = 12.7 *p* = 0.004, $\eta_{\text{p}}^{2}$: 0.52), time (*F*_(1,12)_ = 90.9 *p* = 0.001, $\eta_{\text{p}}^{2}$: 0.88), and interaction effect (*F*_(1,12)_ = 172.1 *p* = 0.001, $\eta_{\text{p}}^{2}$: 0.94) for timed up and go test. In addition, there was a significant main group (*F*_(1,12)_ = 29.0 *p* = 0.001, $\eta_{\text{p}}^{2}$: 0.71), time (*F*_(1,12)_ = 54.6 *p* = 0.001, $\eta_{\text{p}}^{2}$: 0.82), and interaction effect (*F*_(1,12)_ = 41.1 *p* = 0.001, $\eta_{\text{p}}^{2}$: 0.77) for chair stand.

### Brain Oscillation

**Figure 3:**
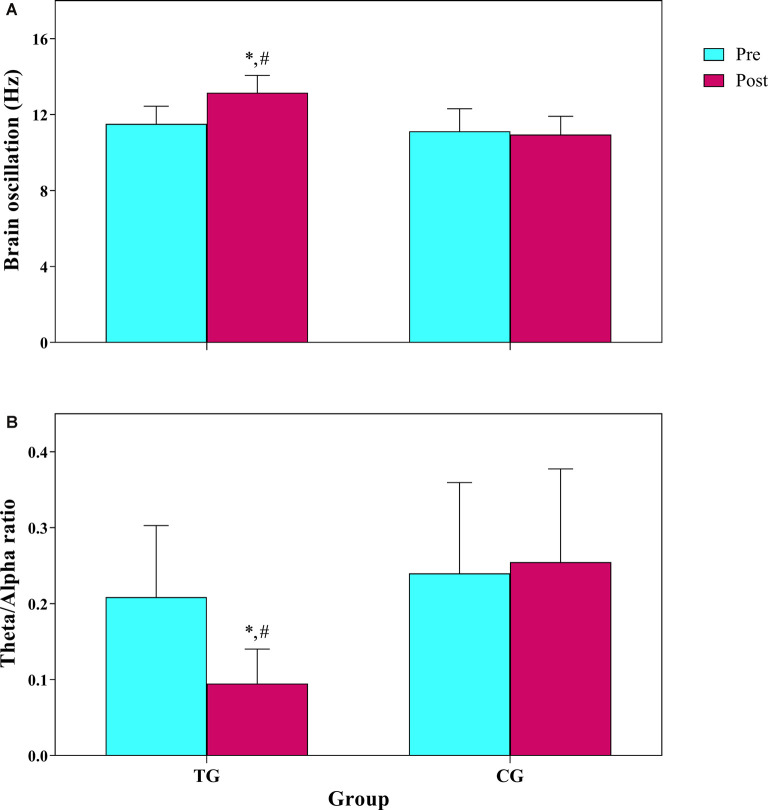
**(A)** Frequency of brain oscillation, **(B)** theta/alpha ratio in 10 min resting EEG. TG, training group; CG, control group. *The significant difference with pre-test. ^#^The significant difference between groups.

Following 12 weeks of combined training, the percentage of resting average frequency of brain oscillation in occipital region in the TG increased significantly by 14.5%; change from alpha range to beta frequency (11.51 to 13.15 Hz), but there was no significant change (−1.4%) in control group (11.13 to 10.95 Hz). Descriptive statistics of the brain oscillation are presented in Table 1. The results of repeated measure ANOVA showed there was a significant main group (*F*_(1,12)_ = 11.4 *p* = 0.005, $\eta_{\text{p}}^{2}$: 0.48), time (*F*_(1,12)_ = 63.7 *p* = 0.001, $\eta_{\text{p}}^{2}$: 0.84), and interaction effects (*F*_(1,12)_ = 39.7 *p* = 0.001, $\eta_{\text{p}}^{2}$: 0.77) for resting average frequency of brain oscillation (Figure 3). We found no significant main group (*F*_(1,12)_ = 3.2 *p* = 0.098, $\eta_{\text{p}}^{2}$: 0.21) and time (*F*_(1,12)_ = 3.6 *p* = 0.080, $\eta_{\text{p}}^{2}$: 0.23) effect, though a significant interaction effect existed (*F*_(1,12)_ = 6.7 *p* = 0.024, $\eta_{\text{p}}^{2}$: 0.36) for percentage of alpha oscillation. While for percentage of beta oscillation, a significant main group (*F*_(1,12)_ = 19.2 *p* = 0.001, $\eta_{\text{p}}^{2}$: 0.62), time (*F*_(1,12)_ = 77.2 *p* = 0.001, $\eta_{\text{p}}^{2}$: 0.86), and interaction effect (*F*_(1,12)_ = 82.1 *p* = 0.001, $\eta_{\text{p}}^{2}$: 0.87) was observed. For percentage of theta oscillation, there was a significant main group (*F*_(1,12)_ = 14.7 *p* = 0.002, $\eta_{\text{p}}^{2}$: 0.55), time (*F*_(1,12)_ = 39.5 *p* = 0.001, $\eta_{\text{p}}^{2}$: 0.77), and interaction effect (*F*_(1,12)_ = 46.2 *p* = 0.001, $\eta_{\text{p}}^{2}$: 0.79). There was a significant main group (*F*_(1,12)_ = 10.5 *p* = 0.007, $\eta_{\text{p}}^{2}$: 0.47), time (*F*_(1,12)_ = 29.1 *p* = 0.001, $\eta_{\text{p}}^{2}$: 0.71) and interaction effect (*F*_(1,12)_ = 33.7 *p* = 0.001, $\eta_{\text{p}}^{2}$: 0.74) for theta/alpha ratio (Figure 3).

### Correlations

Table 2 presents the bivariate Pearson’s correlation coefficient (*r*) between the percentage change of performance parameters and MoCA and GDS. In general, there were moderate to large, positive correlations between MoCA changes and performance induces. Moderate, negative correlations were found between changes in GDS and performance indices. Also, MoCA correlated negatively with the theta/alpha ratio, while GDS correlated positively.

**Table 2:** Pearson’s correlation coefficient between the variables.

		Handgrip	Knee extension	Biceps curl	30 s stand-up	Timed Up and Go test	6 min walking	Chair sit and reach	Theta/alpha ratio
MoCA	*r*	0.81	0.78	0.63	0.68	−0.68	0.36	0.74	−0.56
	*p*	0.001	0.001	0.001	0.001	0.001	0.067	0.001	0.003
GDS	*r*	−0.50	−0.55	−0.61	−0.203	0.56	−0.57	−0.47	0.62
	*p*	0.010	0.004	0.001	0.319	0.003	0.003	0.016	0.001

### Exercise Monitoring

**Figure 4:**
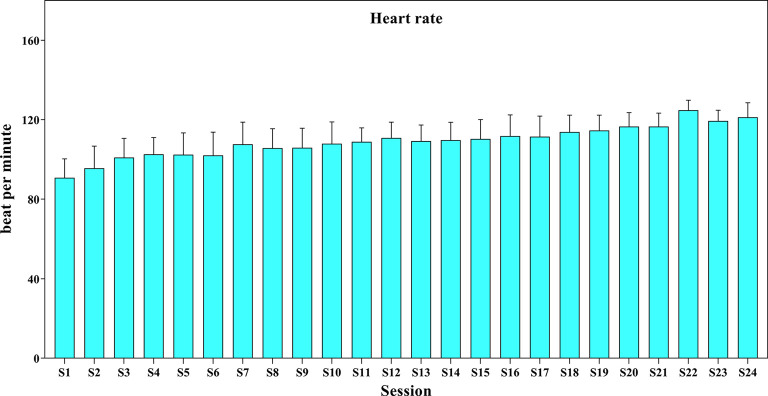
The heart rate (beat/min) during the training sessions.

The mean (SD) of HR during the intervention period was presented in Figure 4. The training began at 50% of maximal HR and reached 70% of maximal HR toward the end of the intervention. The range of HR was 80–125 beat per minute.

## Discussion

This study aimed to evaluate the efficacy of a 12-week of combined training intervention with visual stimulation on the frequency of brain oscillation, cognitive status, and physical performance of patients with AD. The results revealed that following the intervention, patients in the TG group experienced significant improvements in cognitive function, particularly short-term and working memory, attention, and executive function. We also found significant improvements in the depression status of the TG group, compared to CG.

Moreover, significant improvements were observed in the overall physical performance of the participants. These improvements were paralleled with the reduction of the theta/alpha ratio, suggesting that the intervention was effective in involving and activating neurons. Also, moderate to relatively strong correlations were observed between cognitive and performance indices. The findings of our study revealed that the combination of exercise training with mental challenges (such as closing the eyes, attending to auditory stimuli, and trying to control balance by relying on proprioceptive receptors) can be used to improve the independence of patients with AD.

Cognitive impairments, including memory, speech, attention, and executive function impairments, are among the characteristics of AD, which can be measured with the MoCA test in this population. In our study, after the intervention, cognitive performance (the MoCA test) improved with a large effect size (28.4%; $\eta_{\text{p}}^{2}$ = 1.7). Improvements were observed in short-term memory, executive function, attention, and working memory. Also, since closing eyes activate different areas of the brain, in particular the hippocampus (Ben-Simon et al., 2008), which plays roles in spatial memory, balance, and concentration (Rubin et al., 2014), and since our subjects had to close their eyes during the training protocol, our results might be related to hippocampal activation. However, we have not demonstrated this in our study and suggest it for further investigation in this area. Our results are in agreement with previous research, supporting the protective effects of physical training on cognitive function (Burns et al., 2008; Morris et al., 2017).

Although the exact mechanisms of the protective effect of exercise training on the mental health of AD patients are less clear, several mechanisms have been proposed, including the increase of blood supply to the brain, improvement of metabolic health, production of neurotrophic factors (Gallaway et al., 2017), increased size of the hippocampus (Erickson et al., 2011), and increasing gray and white matter volumes in the inferior parietal cortex and the hippocampus over a long period (Burns et al., 2008; Voss et al., 2013). These alterations are associated with memory and cognitive performance, as well as changes in the power of brain oscillation. In this regard, a previous study showed that even a 12-week period of aerobic training could expedite neuroplasticity and promote brain health in sedentary adults (Chapman et al., 2013); the observed improvements in brain function were attributed to the increased physical activity of the participants.

Depression is one of the most common symptoms and consequences of AD, which exacerbates the negative consequences of this disease. Research has shown that regular exercise training in the short-term had obvious effects on depression management (Craft and Perna, 2004). The results of the present study also demonstrated the effectiveness of combined training in reducing depression. Based on the results, depression was inversely correlated with physical fitness indices and positively correlated with the theta/alpha ratio. Several mechanisms can justify the positive effects of exercise training on depression. Improvement of independence, daily life activities, and the mood is among the advantages of exercise training for reducing depression. Also, social interaction between participants in the TG during the training period was effective in improving mood and managing depression.

Moreover, exercise-mediated production of neurotransmitters, such as dopamine, serotonin (Paillard et al., 2015), and brain-derived neurotrophic factor (Wang and Holsinger, 2018), contributes to the treatment of depression. Also, AD-induced high cortisol levels exert neurotoxic effects on the hippocampus and promote oxidative stress, leading to depression, neurodegeneration, and cognitive decline (Ouanes and Popp, 2019). On the other hand, one of the protective effects of regular exercise is lowering the serum cortisol level (Corazza et al., 2014). Although these factors were not measured in this study, the observed improvements can be explained by these mechanisms.

Researches showed that the changes in the ratio of alpha, beta, and theta oscillations are the AD markers, so we extracted the data of these brain oscillations. On the other hand, we did not consider the gamma and delta oscillations, because the delta and gamma oscillations are activated during sleep and cognitive learning activities, respectively (Abhang et al., 2016). The resting alpha and beta oscillation indicate relaxed and alert wakefulness (Abhang et al., 2016), and the theta/alpha ratio is indicative of cognitive deficits (Fahimi et al., 2017). Decreased alpha oscillation power has been reported in AD (Hsiao et al., 2013; Koelewijn et al., 2017), which is associated with an increase in the theta/alpha ratio. Therefore, the reduction of theta/alpha ratio in our study suggests that a combined training period with mental challenges for AD patients activates the mechanisms in the brain, which improve cognitive processing. This finding is in line with a previous study, which showed that 10 weeks of limb exercise significantly increase the alpha and beta oscillation power in all brain areas of older adults with MCI (Jiang et al., 2019); however, this study did not report the theta/alpha ratio.

Although the exact mechanisms of change in the brain oscillation ratio due to exercise training are unknown in AD, the alpha oscillation power seems to be correlated with higher cerebral blood flow in the brain areas, involved in attentional modulation (Jann et al., 2010). Alpha oscillations are generated mainly in the occipital and parietal lobes, as well as thalamocortical feedback loops, whereas beta oscillations mainly originate from the frontal and temporal lobes (Abhang et al., 2016). The eye-closing part of our training protocol forced the individuals to focus on the auditory and proprioceptive data, originating from beta and alpha oscillation.

Moreover, the sensory data are distributed in different areas of the cortex through the thalamus. Therefore, our intervention was highly effective in activating the brain parts involved in attention. In contrast, Gutmann et al. (2015) were reported no changes in alpha oscillation power after 4 weeks of moderate exercise training. It seems that methodological differences can explain these contradicting results. The subjects of the latter study were healthy young men, while the populations of our study were older AD adults. Overall, the findings demonstrated that 12 weeks of training combined with mental challenge reduced the theta/alpha ratio by improving the neurophysiological mechanisms.

AD is associated with the loss of muscle mass and strength, reduced balance, and reduced cardiovascular fitness, leading to inability to perform daily activities, loss of independence, and poor quality of life (Santana-Sosa et al., 2008; Burns et al., 2010; Lane et al., 2018) therefore, our subjects’ baseline fitness level was very poor. Our findings showed that 3 months of combined training caused substantial improvements in the performance indices. Resistance exercises (dumbbells, TheraBand, and rubbers) led to increased strength and maintenance of muscle mass, balance exercises (walking on a beam board) and eyes-closed exercises improved proprioception, and consecutive exercises led to increased cardiovascular fitness.

Improved balance in the present study is especially important, as balance and mobility impairments in AD patients are associated with the risk of falling and reduced quality of life. It is worth mentioning that the observed improvements after exercise training are not population-specific, as comparable increments have been observed in the physical capacity of other populations after a short-term training program (de Vreede et al., 2005). Improved fitness components appear to be correlated with the ability to perform daily tasks and quality of life. This finding is in line with a previous study, which examined the effects of exercise training on functional capacity in AD patients (Santana-Sosa et al., 2008).

Santana-Sosa et al. (2008) demonstrated that a 12-week combined training program led to significant improvements in the upper and lower body muscle strength, endurance fitness, balance, and ability to perform daily activities. Also, moderate-to-large positive interactions were observed between changes in physical parameters and cognitive function. Moreover, there was a strong association between the change of muscle strength (especially handgrip) and MoCA. This finding was supported by a previous study, which showed a strong relationship between muscle atrophy and declined cognitive function (Burns et al., 2010). Kim et al. (2019) also reported a positive relationship between the handgrip strength and cognitive function of elderly Korean adults. Moreover, Burns et al. (2008) reported that increased cardiorespiratory fitness is associated with reduced brain atrophy in AD patients. Based on the findings, exercise training can be an important adjunct to the pharmacological treatment of AD.

We acknowledge that there are some limitations to this study. First, the posttest date coincided with the pandemic of COVID-19 in Iran, and we lost some of our participants. Second, due to the lack of full-time caregivers, transportation was difficult, and the workout time was not consistent; however, all participants completed 24 workout sessions. Third, we did not have access to quantitative EEG; therefore, we suggest using structural and functional brain imaging to assess quantitative changes in the brain structure and function in the future. Finally, we did not determine the period when these adaptations remained constant, which indicates the importance of follow-up after 3, 6, or even 12 months.

## Conclusions

In conclusion, a 12-week combined training program, including resistance, balance, and cardiovascular exercises with closed-eyes stimulation, improved the performance capacity of patients with AD. Also, this intervention improved brain health and activated neurophysiological mechanisms, which are associated with increased cognitive function and decreased theta/alpha ratio. Moreover, our findings supported the hypothesis that cognitive functions are correlated with muscle strength-related physical fitness in patients with AD.

## Data Availability Statement

The original contributions presented in the study are included in the article, further inquiries can be directed to the corresponding author.

## Ethics Statement

The studies involving human participants were reviewed and approved by the Ethic committees for Sport Sciences Research Institute of Iran with code IR.SSRI.REC.1398.037 and were conducted in accordance with the Declaration of Helsinki. The study has been registered in the Iranian Registry of Clinical Trials (IRCT) with registration number: IRCT20190504043468N1. The patients/participants provided their written informed consent to participate in this study.

## Author Contributions

SA-S and EP conceived the study. EP, FM, and BT conducted the experiments. SA-S and MB analyzed the study. SA-S, FM, and MB interpreted the data for the study. All authors made substantial contributions to the design of the work, drafted the work or revised it critically for important intellectual content, provided final approval of the version to be published, and agreed to be accountable for all aspects of the work in ensuring that questions related to the accuracy or integrity of any part of the work are appropriately investigated and resolved. All authors read and approved the final manuscript. All authors contributed to the article and approved the submitted version.

## Conflict of Interest

The authors declare that the research was conducted in the absence of any commercial or financial relationships that could be construed as a potential conflict of interest.

## Abbreviations

1-RM: one-repetition maximum
6MWT: six-minute walk test
AD: Alzheimer’s disease
ANOVA: analysis of variance
BF: body fat
BMI: body mass index
CG: control group
CSR: chair sit and reach
CV: coefficient of variation
EEG: electroencephalography
ES: effect size
GDS: geriatric depression scale
HR: heart rate
IC: internal consistency
ICC: intra-class correlation coefficient
MCI: mild cognitive impairments
MoCA: montreal cognitive assessment
SD: standard deviation
TG: training group
TUG: timed up and go.
